# Analysis of the factors related to academic disapproval in the education of nurses: A mixed-method study*

**DOI:** 10.1590/1518-8345.4458.3411

**Published:** 2021-04-12

**Authors:** George Oliveira-Silva, Natália Del’ Angelo Aredes, Hélio Galdino-Júnior

**Affiliations:** 1 Universidade Federal de Goiás, Goiânia, GO, Brazil.; 2 Scholarship holder at the Coordenação de Aperfeiçoamento de Pessoal de Nível Superior (CAPES), Brazil.; 3 Universidade Federal de Goiás, Faculdade de Enfermagem, Goiânia, GO, Brazil.

**Keywords:** Nursing Students, Nursing Education, Academic Success, Academic Failure, Academic Performance, Nursing, Estudantes de Enfermagem, Educação em Enfermagem, Sucesso Acadêmico, Fracasso Acadêmico, Desempenho Acadêmico, Enfermagem, Estudiantes de Enfermería, Educación en Enfermería, Éxito Académico, Fracaso Escolar, Rendimiento Académico, Enfermería

## Abstract

**Objective:**

to evaluate the frequency and factors associated to disapproval among nursing students.

**Method:**

a descriptive and cross-sectional study, outlined by the convergent mixed method. A total of 88 nursing students of a Brazilian public university took part. The *Reduced Academic Experiences Questionnaire* was used for evaluating academic adaptation. The association of the variables in the study with disapproval was verified by bivariate analysis.

**Results:**

the frequency of disapprovals in the sample was 68.2%, recurrences in the same discipline in 39.8%, with the associated factors: age over or equal to 22 years old (p=0.015), family income below 2 minimum wages (p=0.019) and lag in the curricular flow (p<0.001). Disciplines with higher frequencies of disapprovals are of the basic area, taught in the first two years of the course and common to the health courses. Students without disapprovals had better perception of physical and psychological well-being (p=0.002), good interpersonal relationships (p=0.017) and more assertive study behaviors (p=0.005). Personal, study-related and institutional issues were motivating.

**Conclusion:**

the results reveal a high rate of disapproval, especially in the basic area. An association was found between disapprovals and mental health for nursing students during their education process, and difficulties were pointed out that can culminate with the disapproval rate in the curricular flow.

## Abstract (pt)

**Objetivo:**

avaliar a frequência e fatores associados à reprovação entre estudantes de enfermagem.

**Método:**

estudo descritivo, transversal, delineado pelo método misto convergente. Participaram 88 estudantes de enfermagem de uma universidade pública brasileira. Para avaliação da adaptação acadêmica utilizou-se o *Questionário de Vivências Acadêmicas Reduzido*. A associação das variáveis do estudo com a reprovação foi verificada por análise bivariada.

**Resultados:**

a frequência de reprovações na amostra foi de 68,2%, reincidentes na mesma disciplina em 39,8%, com os fatores associados: idade maior ou igual a 22 anos (p=0,015), renda familiar inferior a 2 salários mínimos (p=0,019) e defasagem no fluxo curricular (p<0,001). Disciplinas com maiores frequências de reprovações são da área básica, ministradas nos dois primeiros anos do curso e comuns aos cursos da saúde. Estudantes sem reprovações apresentaram melhor percepção de bem-estar físico e psicológico (p=0,002), bom relacionamento interpessoal (p=0,017) e comportamentos de estudo mais assertivos (p=0,005). Questões pessoais, relacionadas ao estudo e institucionais foram motivadores apontados.

**Conclusão:**

os resultados revelam alto índice de reprovações, sobretudo na área básica. Foi encontrada associação entre reprovações e saúde mental dos estudantes de enfermagem durante seu processo de formação, e foram apontadas dificuldades que podem culminar com a taxa de insucesso no fluxo curricular.

## Abstract (es)

**Objetivo:**

evaluar la frecuencia y los factores asociados a la reprobación entre estudiantes de Enfermería.

**Método:**

estudio descriptivo y transversal, delineado por el método mixto convergente. Participaron 88 estudiantes de Enfermería de una universidad pública brasileña. Para evaluar la adaptación académica se utilizó el Cuestionario de Experiencias Académicas Reducidas. La asociación de las variables de estudio con la reprobación se verificó mediante análisis bivariado.

**Resultados:**

la frecuencia de reprobaciones en la muestra fue del 68,2%, siendo el 39,8% recurrente en la misma asignatura, con los siguientes factores asociados: edad mayor o igual a 22 años (p=0,015), ingreso familiar por debajo de 2 salarios mínimos (p=0,019) y desfase en el flujo curricular (p<0,001). Las asignaturas con mayor frecuencia de reprobación se encuentran en el campo básico, se imparten en los dos primeros años del curso y son comunes a los cursos de salud. Los estudiantes sin reprobaciones tuvieron una mejor percepción del bienestar físico y psicológico (p=0,002), buenas relaciones interpersonales (p=0,017) y conductas de estudio más asertivas (p=0,005). Cuestiones personales, relacionadas con el estudio e institucionales fueron motivadores señalados.

**Conclusión:**

los resultados muestran un elevado índice de reprobaciones, especialmente en el campo básico. Se encontró una asociación entre reprobaciones y la salud mental de los estudiantes de Enfermería durante su proceso de formación, y se señalaron dificultades que pueden culminar en el índice de reprobaciones en el flujo curricular.

## Introduction

The phenomena of disapprovals and dropout in higher education still occur in expressive dimensions in Brazil^(^ 1 ^)^. Sociodemographic conditions and personal influences affect values, expectations and aspirations of the students, intervening in the decision to remain or to drop out^(^ 2 ^-^ 3 ^)^, besides the aspects inherent to the educational process that may be different from those that are desirable by the students.

In nursing education, studies point out that socioeconomic factors of the enrolled individuals, such as low financial condition, insufficient basic education and difficulties related to the abilities to process information, to solve problems and to reason logically, can have a relation with low academic performance^(^ 3 ^-^ 4 ^)^ while, in different realities, differences in the performance depending on the students’ previous education^(^ 5 ^)^ are verified, drawing the attention to the challenges experienced by the universities today.

Nurses are essential for strengthening health care in Brazil and worldwide, and the need is clear for more professionals available to act in face of the growing demands of the world population^(^ 6 ^)^. Thus, strategies must be created within the teaching institutions to ensure the education of qualified, critical and committed professionals to act in the health system, avoiding dropouts and retentions in this process.

For this purpose, education scholars have been engaged in discussing pedagogical proposals, methods and strategies focused on the students, aiming at their autonomy and active participation in their own learning process^(^ 7 ^)^. Furthermore, the literature denotes the efforts of the health and education areas in the qualification of professionals who are articulated among the different categories, who navigate among the disciplines and focus on integral and integrated assistance to the users of the Unified Health System^(^ 7 ^-^ 8 ^)^.

When planning the training of nurses, according to the National Curricular Guidelines, it is necessary to consider that the variations in academic performance are of a multi-factorial order and have important implications for the course and completion of undergraduate studies. Nevertheless, intercurrences such as academic disapproval are related to the appearance of psychosomatic diseases in nursing students^(^ 9 ^-^ 10 ^)^, issues which deserve to be highlighted in view of the important prevalence of the depressive symptoms found in this population today^(^ 11 ^)^.

This theme is widely discussed in the international setting^(^ 5 ^,^ 12 ^-^ 13 ^)^; however, in Brazil, few studies approach academic disapproval among nursing students and its related factors^(^ 14 ^-^ 15 ^)^. In general, the studies evaluate these metrics in a macro way as institutional indicators of management in higher education^(^ 16 ^-^ 17 ^)^, not approaching the particularities that allow deepening the understanding of the phenomenon. Thus, this study was carried out in view of the gap in the literature about disapprovals, especially in nursing education.

Understanding the factors that interfere in the disapprovals of nursing students can help in the development of support strategies, with a view to improve quality in the education of nurses. Thus, the study objectives were to evaluate the frequency and factors associated with disapproval among nursing students, in addition to identifying factors that influence this occurrence in their perspective.

## Method

This is a descriptive and cross-sectional study, delineated by the convergent mixed method, in which the quantitative and qualitative data were collected concomitantly^(^ 18 ^)^, with associated analysis in the data interpretation stage, starting from the presentation of the results combined from the information obtained in both stages of data collection. The mixed methods approach was used to better understand the disapproval phenomenon, valuing both the generalization of the findings and the depth of the interpretation.

Data was collected between April 2018 and March 2020 with nursing students from a public Higher Education Institution (HEI) of the Brazilian Midwest. The studied institution foresees a minimum duration for the course of 5 years (10 semesters), full-time with a total workload of 4,396 hours and currently has 284 active students in the course. Its pedagogical proposal is interdisciplinarity and articulation between theory and practice, and its curricular components are structured in common core disciplines, referring to the basic area of Health Sciences; specific core disciplines, referring to the Nursing components; besides optional disciplines; free core disciplines (which can be freely performed by the student in any academic unit of the university) and complementary activities.

The inclusion criteria for participating in the study were the following: being an undergraduate nursing student, having entered between 2010 and 2016, and having attended at least two years of undergraduate studies. Exclusion criteria: students who entered the course by transfer, because they could not represent the full experience of nursing undergraduate in the institution studied, considering the use of credits and disciplines in the transition between HEIs.

Based on the prevalence of academic disapprovals pointed in a recent study with the same theme^(^ 13 ^)^, the sample calculation was made to estimate the amount of participants needed for the study. Considering a 95% confidence level, 5% error and 10% possible losses, a minimum sample of 80 students was estimated.

At the data collection time, the institution had a universe of 154 students able to participate in the study and who received the online form sent by e-mail. Of these, 88 students from the nursing course answered the online form, representing 57.1% of the total. Despite the challenge of collecting data by means of online questionnaires in terms of adherence to the target population, the answer rate obtained in this study was higher than in other studies that used electronic questionnaires for collecting data from university students^(^ 19 ^-^ 20 ^)^.

The questionnaire was structured to address sociodemographic and academic data, and was adjusted after a pilot data collection with 12 students. Besides the closed questions, the questionnaire had an open question for students with disapprovals about the factors to which they attributed this occurrence.

Due to the importance of understanding the variables that interfere with academic performance in the university context, the study used the *Reduced Academic Experience Questionnaire* (QVA-r)^(^ 21 ^-^ 22 ^)^, an instrument adapted and validated for the Brazilian reality under study with 626 university students, obtaining good levels of reliability in all the dimensions (α>0.7)^(^ 22 ^)^.

The instrument has 55 questions in a Likert scale ranging from 1 to 5 and is divided into five dimensions that aim to analyze the following: personal dimension (perceptions of physical and psychological well-being); interpersonal dimension (relationship and establishment of bond with peers); course/career dimension (adaptation to the course and career perspective); study dimension (study skills, habits and time management); and institutional dimension (adaptation to the institution, use of resources provided by it and perception of the quality of services)^(^ 21 ^)^.

The statistical analysis was performed with the *Statistical Package for the Social Science* (SPSS), version 25.0. The variables were described in the form of frequencies, percentages, means and standard deviation. The Cronbach›s alpha coefficient was used to evaluate the internal consistency of QVA-r. After Kolmogorov-Smirnov normality test, for the parametric variables we adopted the Student›s t test and, for the non-parametric, the Mann-Whitney test. For the association of qualitative variables, the Pearson›s Chi-square and Fisher’s Exact tests were used. In all the analyses, a 95% confidence level (p<0.05) was established.

The analysis of the qualitative data was carried out by the content analysis modality^(^ 23 ^)^ and, for the elaboration of the categories and thematic axes, the bases of the QVA-r^(^ 21 ^)^ were used as conceptual framework. The sense-related cores expressed during the communication process were identified, being followed the following stages: pre-analysis, exploration of the material, treatment of the results obtained, and interpretation. The analysis procedures were carried out by two researchers in order to mark the follow-up related to the stages and the final result. To designate each speech fragment, preserving the anonymity of the participants, we selected the term “Student”, described by the letter “E” (“*Estudante*” in Portuguese), followed by Arabic numerals according to the order of analyzed answers of the complete questionnaires.

The research project obtained a favorable opinion from the research ethics committee (number 2,446,291) and all the stages of the study respected CNS Resolution number 466/2012.

## Results

A total of 88 nursing students took part in the quantitative stage of the study. As for their profile, the mean age of the sample was 23.02 years old (± 2.69), with predominance of female participants (94.3%; n=83), single (89.7%; n=79), not white-skinned (52.2%; n=46) and living with their parents or spouses at the time of the survey (69.3%; n=61). 13.6% (12) of the sample reported working, and the relative frequency of dedication was up to 20 hours *per* week (8%; n=7), followed by 21 to 39 hours *per* week (2.3%; n=2) and over 40 hours *per* week (3.4%; n=3).

As for the year of entry, 1.1% (n=1) entered in 2011, 6.8% (n=6) in 2012, 22.7% (n=20) in 2013, 26.1% (n=23) in 2014, 19.3% (n=17) in 2015 and 23.9% (n=21) in 2016; a distribution that allowed for the analysis of the possibility for disapproval in common or specific core disciplines throughout the course, with more than one third of the sample completing supervised internship (final phase of the course).

The majority (68.2%; n=60) showed disapprovals throughout the academic course, and 39.8% (n=35) presented more than one disapproval *per* discipline. Regarding the academic path, it was observed that only 32.5% (n=27) of the sample followed the flow of disciplines suggested by the institution, while a considerable portion was lagging in the curricular flow (67.5%; n=56), emphasizing the pre-requisites locks.

Regarding the perception of academic performance, 29.5% (n=26) evaluated themselves with great performance, 48.9% (n=43) evaluated themselves with good performance, 15.9% (n=14) classified themselves as having regular performance, while 5.6% (n=5) attributed bad performance.

The analysis of the factors associated to disapprovals showed that the age (PR=0.69; CI 95%: 0.50-0.95; X^2^=5.87; p=0.01) and lag in the curricular flow (PR=9.0; CI 95%: 3.09-26.15; X^2^=70.0; p<0.001) variables were predictors, with age less than or equal to 22 years old being a protection factor (Table 1).

**Table 1:** Association of the study variables with the number of disapprovals among nursing students from a Public Higher Education Institution in the Midwest of Brazil, 2020 (N = 88)

Variables	N (%)	Disapprovals		PR* (95% CI^†^)	*p*-value^‡^
		Yes (%)	No (%)		
Gender					
Female	83 (94.3)	58 (69.9)	25 (30.1)	1.74 (0.59-5.15)	0.321^§^
Male	5 (5.7)	2 (40.0)	3 (60.0)		
Age					
≤ 22 years old	40 (45.5)	22 (55.0)	18 (45.0)	0.69 (0.50-0.95)	0.015
≥ 23 years old	48 (54.5)	38 (79.2)	10 (20.8)		
Marital status					
Single	79 (89.8)	53 (67.1)	26 (32.9)	0.86 (0.58-1.26)	0.713^§^
Married/Stable union	9 (10.2)	7 (77.8)	2 (22.2)		
Skin color					
White	42 (47.7)	35 (76.1)	11 (23.9)	1.27 (0.94-1.72)	0.096
Non-white	46 (52.3)	25 (59.5)	17 (40.5)		
Living with					
Parents/Spouse	61 (69.3)	45 (73.8)	16 (26.2)	1.32 (0.91-1.92)	0.091
Others	27 (30.7)	15 (55.6)	12 (44.4)		
Work					
Yes	12 (13.6)	10 (83.3)	2 (16.7)	0.78 (0.58-1.06)	0.324^§^
No	76 (86.4)	50 (65.8)	26 (34.2)		
Family income					
Up to 2 MWs^||^	26 (30.6)	21 (77.8)	6 (22.2)	1.24 (0.93-1.64)	0.166
Over 2 MWs^||^	59 (69.4)	37 (62.7)	22 (37.3)		
High School					
Public school	47 (55.3)	34 (72.3)	13 (27.7)	1.14 (0.84-1.54)	0.366
Private school	38 (44.7)	24 (63.2)	14 (36.8)		
Entrance					
ENEM^¶^	39 (45.3)	26 (66.7)	13 (33.3)	0.94 (0.71-1.26)	0.724
Admission test	47 (54.7)	33 (70.2)	14 (29.8)		
Affirmative Action Admission**					
Yes	37 (42.0)	25 (67.6)	12 (32.4)	0.98 (0.73-1.31)	0.916
No	51 (58.0)	35 (68.6)	16 (31.4)		
Lag in the curriculum flow					
Yes	56 (67.5)	56 (100)	0 (0.0)	9.0 (3.09-26.15)	<0.001
No	27 (32.5)	3 (11.1)	24 (88.9)		

* PR = Prevalence ratio;

† CI = Confidence interval;

‡ *p*-value = Pearson's Chi-square Test;

§ Fisher's Exact Test;

|| MWs = Minimum wages;

¶ ENEM =*Exame Nacional do Ensino Médio*(National High School Exam);

** Public policy regulated by the Law 12,711/2012 for the allocation of vacancies for entry into Higher Education to students from minority groups, coming from public schools with a family income below 1.5 minimum wages or self-declared black, brown or indigenous students

When compared by the disapproval distribution *per* student, the age group and family income variables were significant, suggesting that students aged 22 years old or less (U=566.00; p=0.003) and with an income above 2 minimum wages (U=508.00; p=0.019) have fewer disapprovals.

The frequency of disapprovals *per* discipline is shown in Table 2. It is important to note that the specific nursing disciplines represent only 14.9% of the reported disapprovals, whose total was 187 in this sample. Thus, the main challenge is contained in the disapprovals in the common core disciplines of the health area.

**Table 2:** Number of disapprovals by discipline among nursing undergraduates of a Public Higher Education Institution in the Midwest, Brazil, 2020 (N = 60)

DISCIPLINES	N	%
*Common core disciplines*		
Pathology	32	53.3
Biochemistry	25	41.6
Physiology	20	33.3
Basic Pharmacology	14	23.3
Histology	12	20.0
Parasitology	11	18.3
Biophysics	10	16.6
Organ Histology	9	15.0
Genetics	8	13.3
Human Anatomy 1	6	10.0
Immunology	4	6.6
Human Anatomy 2	3	5.0
Applied Pharmacology	2	3.3
Microbiology	2	3.3
Sociology	1	1.6
*Core-specific disciplines*		
Clinical Nursing	10	16.6
Bases for Caring for the Individual and the Family 1	7	11.6
Bases for Caring for the Individual and the Family 2	5	8.3
Infectious Disease Nursing	3	5.0
Pre-Hospital Care	1	1.6
Epidemiology	1	1.6
Biological Risk and Biosafety	1	1.6

Of the total sample, up to the year 2019, 67 students would be able to complete the course within the minimum expected time of five years, and 28.4% (n=25) effectively completed this period. Of those retained, 23.8% (n=21) completed it in six years, 6.8% (n=6) in seven years, and 1.1% (n=1) in eight years. Among those who completed it in six years, only 1.1% (n=1) had a delay in training due to enrollment lock. Still, 15.9% (n=14) remained in retention in the course. Whereas the disapprovals are the main drivers of the delay in training and excluding those who locked the course, students took a mean of 5.6 years to complete the course (minimum 5; maximum 8).

The results of QVA-r revealed that the sample has good academic adaptation. The instrument obtained adequate internal consistency in the verification of academic adaptation, both in the dimensions and in the total score, except for the Institutional dimension, which displayed low internal consistency. The independence *t* test showed that there was no difference in the academic adaptation of students with or without disapprovals. However, in the analysis by dimensions, students without disapprovals obtained better scores in the personal, interpersonal and study dimensions (Table 3).

**Table 3:** Association of the occurrence of disapprovals with the mean in QVA-r* for nursing students of a Public Higher Education Institution in the Midwest, Brazil, 2020 (N=88)

Dimensions	Mean	α^†^	Disapprovals		*t§* ^§^test	*p*-value
			Yes (SD^‡^)	No (SD^‡^)		
Total score	3.53	0.90	3.46 (±0.45)	3.66 (±0.50)	t(86)=-1.92	0.058
Personal	2.87	0.89	2.68 (±0.76)	3.27(±0.89)	t(86)=-2.43	0.002
Interpersonal	3.20	0.87	3.08 (±0.63)	3.45 (±0.69)	t(86)=0.93	0.017
Career/Course	3.90	0.84	3.94 (±0.66)	3.80 (±0.62)	t(86)=-2.89	0.353
Study	3.24	0.83	3.07 (±0.74)	3.57 (±0.76)	t(86)=0.85	0.005
Institutional	4.29	0.65	4.32 (±0.50)	4.22 (±0.44)	t(86)=-1.83	0.397

* QVA-r =*Questionário de vivências acadêmicas reduzido*(Reduced academic experience questionnaire);

† α = Cronbach›s Alpha;

‡ SD = Standard deviation;

§ Student's t test

To better understand the association between disapproval and study the behaviors adopted by the students, the prevalence ratios were checked and Pearson’s chi-square test was performed with the answers to the questions of the study dimension of QVA-r. Thus, the answers were transformed into a 3-point Likert type scale (Table 4).

**Table 4:** Behaviors of nursing students of a Public Higher Education Institution in the Midwest identified in the Study dimension of QVA-r*, Brazil, 2020 (N=88)

Variables	Yes (%)	Sometimes (%)	No (%)
In my studies I am managing to keep pace with my classmates.	44 (50.0)	21 (23.9)	23 (26.1)
I manage my time well.	19 (21.6)	29 (33.0)	40 (45.5)
I make a daily plan of the things I have to do.	20 (22.7)	26 (29.5)	42 (47.7)
I manage to have academic work always up to date.	33 (37.5)	21 (23.9)	34 (38.6)
I know how to set priorities when it comes to organizing my time.	38 (43.2)	22 (25.0)	28 (31.8)
I take good notes from classes.	47 (53.4)	19 (21.6)	22 (25.0)
I can be effective in my preparation for the tests.	32 (36.4)	34 (38.6)	22 (25.0)
I try to systematize/organize the information given in classes.	41 (46.6)	20 (22.7)	27 (30.7)
I have the ability to study.	59 (67.0)	22 (25.0)	7 (8.0)
I am punctual for the classes.	66 (75.0)	12 (13.6)	10 (11.4)

* QVA-r = Reduced academic experience questionnaire

Therefore, it became evident that following the pace of classmates (PR = 0.60; 95% CI: 0,43-0,83; X^2^=7.90; p=0.005), making good time management (PR=0.51; 95% CI: 0,29-0,88; X^2^=9.91; p=0.002), keeping the academic work always up to date (PR = 0.66; 95% CI: 0,46-0,93; X^2^=6,01; p=0.01), establishing priorities with regard to the organization of time (PR=0.66; 95% CI: 0,45-0,97; X^2^=4.22; p=0.04) and being effective in preparing for the tests (PR=0.60; 95% CI: 0,39-0,93; X^2^=4.99; p=0.02) were protective behaviors against disapprovals.

Of the 60 students with disapprovals, 53 answered the open question “to what factors do you attribute the disapproval?” and were included in the qualitative analysis. The answers were typified and separated according to categories and thematic axes. Through the analysis of the answers, three categories emerged, being related to personal issues, study behaviors, and institutional factors (Figure 1).

**Figure 1:** Analysis categories and thematic axes of the answers from Nursing students of a Public Higher Education Institution in the Midwest, Brazil, 2020

CATEGORIES	THEMATIC AXIS
*Personal*	Maturity
	Work
	Psychological and emotional factors
*Study*	Learning
	Dedication
	Time management
*Institutional*	Professor/Discipline methodology
	Professor-student relationship
	Curricular structure

In the “personal” category, issues emerged that are attributed to disapprovals related to the academic experience, but associated with this perspective, capable of influencing the decision to remain or to drop out from the course. They appear in 92.4% (n=49) of the answers. Generally, they are related to health and can be due to the family relationship. Immaturity was reported as an important factor in the student’s difficulty in committing to the discipline or to the course.

*At that time I went through some problems at home, and with that I was a little dispersed in college, which was during the fourth and fifth period, I even thought about giving up, because due to the disapprovals I lost my scholarship, but I continued because I like the course* (E9, 19 years old, 2 disapprovals).

[...] *some were due to my immaturity as an academic for entering college very young and with some personal/family problems* (E33, 24 years old, 6 disapprovals).

Work was also a variable considered in the analysis, representing a matter of subsistence for some students. In this sense, the full-time course and the need to work represent obstacles for the student to perform both activities successfully.

*Most of the time I needed to skip classes to work, to do some part-time jobs, if I didn’t do them I wouldn’t be able to eat in college* (E8, 21 years old, 5 disapprovals).

*Having to work in the period of the discipline in the case of parasitology, I was doing night shifts[...]* (E19, 24 years old, 2 disapprovals).

According to the reports, emotional and psychological factors are determinant in the student’s performance in the discipline, because the difficulty in dealing with personal issues impact on the difficulty in adopting study behaviors that meet the learning needs. From the students’ perspective, these factors are related to psychosomatic illnesses experienced in the academic path that hinder their academic performance and influence the occurrence of disapprovals.

*Emotional factors, difficulty in concentrating in the studies, lack of routine [...].* (E53, 25 years old, 5 disapprovals)

*Anxiety, emotional wear and tear, being away from the family* (E21, 22 years old, 4 disapprovals).

*Psychological pressure* [...] *anxiety, nervousness* (E36, 27 years old, 1 disapproval).

The “study” category was related to the behaviors that students adopt, present in 67.9% (n=36) of the answers. Although the students recognize the importance of establishing study routines, the difficulty of managing time (p=0.01) and listing priorities (p=0.04) is perceived, as well as reconciling attendance and dedication in multiple disciplines, considering curricular and extracurricular activities.

*Many dense disciplines at the same time, they did not know how to manage the studies* (E46, 23 years old, 2 disapprovals*)*.

*Pathology was more because the discipline is very complicated and I didn’t have time to dedicate myself enough* (E4, 19 years old, 4 disapprovals).

[...] *in our class schedule they have disciplines that are too heavy to be taught at the same time (until halfway through the course, then it gets good), which sometimes makes the students dedicate themselves more to one than the others* (E44, 22 years old, 4 disapprovals).

The student’s deficit in being able to study autonomously is also highlighted, suggesting the learning difficulty as a strong motivator for disapproval. Some students suggest that this learning difficulty comes from a lagged prior education, which directly implies the lack of theoretical support to accompany the other students.

*Already in physiology I had difficulty in keeping up with the teacher’s* [classes] *... because she didn’t have didactics and at that time I wasn’t a self-taught person so I suffered a lot, I studied a lot, but it wasn’t enough* (E42, 22 years old, 3 disapprovals).

[...] *learning difficulty, lack of knowledge coming from high school* [...] (E22, 25 years old, 8 disapprovals).

The “institutional” category appears in 64.1% (n=34) of the answers, in which there were reports on the methodology adopted by the professor in the discipline and the professor-student relationship.

*The difficulty of the discipline, lack of malleability of the professors* [...] (E14, 24 years old, 5 disapprovals).

*In biochemistry, the professor wanted the students to teach her, which generated some stress and strangeness between the professor - students which reflected in very difficult tests* (E42, 22 years old, 3 disapprovals).

In this aspect, the reinforcement through quantitative data points to the need for better concordance of the teaching-learning processes between professors and students, ensuring that they understand the methods and strategies adopted in the context of nursing education with autonomy and critical thinking. In this way, basing this finding on the weaknesses found in the statistical analysis, they will be able to adopt better behaviors for organizing the information offered in classes (p=0.05) and prepare themselves in a more assertive way for the evaluation stages (p=0.02).

## Discussion

Disapproval among nursing students is a complex phenomenon of a multi-factorial nature that implies the risk for academic dropout and to compromise the contingent of qualified nurses available in the labor market^(^ 24 ^)^. Disapprovals resulting from poor academic performance, important predictors are age, gender, work, critical thinking, and self-efficacy^(^ 3 ^)^.

In this study, there was a high frequency of disapproval in the sample (68.2%), results higher than in a study^(^ 13 ^)^ with nursing students (5.6%) from Portugal, Italy, Czech Republic, Slovakia and Slovenia and in another study with students from Italy^(^ 25 ^)^ (22.2%). There are still no studies in Brazil that indicate the frequency of disapprovals among nursing students that allow for the comparison of this finding in the country, which highlights the pioneering nature of this study and its importance for future discussions regarding nursing education.

The following factors were mainly associated with disapprovals: age and lag in the curriculum flow provided by the course’s pedagogical project. However, it is essential to note that the lag in the curriculum flow is an outcome resulting from the occurrence of disapprovals. Some studies point to age as a predictor of academic performance, suggesting that older students tend to fail less^(^ 3 ^,^ 23 ^,^ 26 ^-^ 28 ^)^; however, in this study, it was identified that age less than or equal to 22 years old was a protective factor for the occurrence of disapprovals. In a study carried out in Australia^(^ 4 ^)^, age older than 23 years was a predictor for low academic performance, corroborating with the findings of this study in the Brazilian Midwest.

Thus, late entry into higher education can be associated with more disapproval, perhaps due to the long interval between the learning practices experienced in high school education and entry into higher education. Immaturity related to the difficulty in making decisions regarding study behaviors was a term used in the reports of some students as a possible motivator for disapproval, a finding reinforced by a study conducted in Singapore^(^ 28 ^)^, suggesting that better academic performance and student maturity are related.

Lag in the curriculum flow was also associated with the occurrence of disapprovals, but possibly the relationship stems from the fact that disapprovals result in delay in the course and consequent lag in the flow of disciplines.

Unlike the results found in Italy^(^ 25 ^)^, the comparison of the distribution of disapprovals *per* student showed that students with a family income above 2 minimum wages have fewer disapprovals in their academic path, suggesting that socioeconomic issues can be important predictors of low academic performance and, consequently, of disapprovals. Considering the change in the profile of those entering Brazilian higher education due to the greater democratization of access, especially in the last decade^(^ 1 ^)^, psycho-pedagogical support strategies and permanence policies have been adopted with a focus on this target population.

Other studies^(^ 13 ^-^ 14 ^)^address the occurrence of disapprovals in the first years of undergraduate nursing. This period is fundamental for the consolidation of basic knowledge in the health area, generally formatted in disciplines that are a prerequisite for specific nursing.

In the Nursing courses, disciplines such as Pathology, Biochemistry, Physiology, Histology and Anatomy have high disapproval rates^(^ 14 ^-^ 15 ^)^, drawing the attention to harm mitigation strategies widely discussed in the universities, such as tutoring and psycho-pedagogical support. In this same sense, the deficit in knowledge arising from previous teaching in conjunction with the number of disciplines offered *per* period are important impact factors for disapprovals.

All the common core disciplines with reported disapprovals are taught in the first two years of the course, reinforcing that this period is essential to define the path of the nursing student and to predict the time needed to complete the course, increasing as more disapprovals occur. In addition, it is an alert for the monitoring of students, avoiding course drop outs.

In this study, it was observed that disapprovals occur more frequently in the common core disciplines, that is, those that are transversal to all courses in the health area. Such findings may show difficulty in the transversality of the teaching-learning process and potentially demonstrate the fragmentation of knowledge, considering that the disapproval rate among specific nursing disciplines is significantly lower than in the basic area. However, further studies must be undertaken to identify this relationship and detail the possible variables involved in this phenomenon.

As for academic adaptation, there was no difference between students with or without disapprovals. However, in the personal, interpersonal and study dimensions of QVA-r, students with no disapprovals obtained higher scores. Conceptually, these results suggest that they have a good perception of physical and psychological well-being, good relationships with their classmates, and assertive study behaviors^(^ 21 ^)^.

These results suggest the need to monitor students who fail, as difficulties in academic adaptation are associated with the occurrence of psychological disorders, such as anxiety and depression, in students from various courses^(^ 29 ^)^ and in nursing students^(^ 30 ^)^.

The importance is emphasized of psychological issues as motivators of the disapprovals reported by students, since several studies have shown the prevalence of anxiety and depression in nursing students^(^ 11 ^,^ 31 ^-^ 33 ^)^. Therefore, the process of adaptation to higher education impacts both on the academic trajectory and on the mental health of the student^(^ 30 ^)^.

In addition to the emotional state, the study behaviors reported by the students pointed out that keeping pace with classmates, managing time, keeping up with academic work, setting priorities and preparing effectively for exams constitute protective behaviors against disapprovals. In this sense, psycho-pedagogical support is essential to encourage students to adopt appropriate study behaviors, in addition to developing a greater capacity to deal with psychosomatic issues^(^ 34 ^)^.

Although no association was identified between the type of basic education and the occurrence of disapprovals, some students perceive the deficit in previous education as a motivator for disapprovals, implying learning difficulty in higher education. The literature has identified the type of school as a predictor of academic success, suggesting that students from technical schools tend to present lower performance^(^ 5 ^)^.

To this end, Brazilian universities have already devised strategies for strengthening psycho-pedagogical support for students who have learning difficulties^(^ 35 ^-^ 36 ^)^. Likewise, academic tutoring has been shown to be a strong strategy for establishing a bond between professor and student, favoring the sharing of learning and the reduction of difficulties encountered in the educational process^(^ 34 ^,^ 37 ^)^.

It is noteworthy that strategies to reduce these difficulties, such as the conduction of study groups among students, favor the sharing of knowledge among peers and can provide better performance in undergraduate courses^(^ 38 ^)^.

Corroborating the findings on study behaviors, the students’ reports point to insufficient dedication as associated with the difficulty in establishing study routines and time management. The work variable was not associated with the occurrence of disapprovals; however, based on the reports, it is suggested that in previous situations in which the student needed to reconcile study with work and this caused disapprovals, results corroborated by other authors^(^ 13 ^,^ 25 ^)^ and that need attention from professors and HEI managers regarding the profile of the students and strategies that favor the inclusion of working students in the nursing courses.

The professor-student relationship is a factor that can intervene in academic performance based on the assumption that this interpersonal relationship is part of the process of teaching and learning, of acting at the theoretical-methodological level with the sharing of knowledge, experiences and motivations and guidance at academic level^(^ 39 ^)^. According to the report from some students, difficulties in this relationship resulted in disapprovals.

Together, the methodology used by the professor in the discipline is a factor that interferes with the students’ learning. Active methodologies have positively impacted academic performance and influenced the decrease in the number of disapprovals^(^ 40 ^-^ 41 ^)^; however, it is worth mentioning that part of the students presents difficulties in adapting to this pedagogical model, potentially due to a history of education in the traditional model^(^ 42 ^)^.

In the scientific literature, there are highlights for characteristics that strengthen student satisfaction and good academic performance, namely: professor qualification, application of participatory teaching methodologies, updated didactics and insertion of the students in the practice environment, making them to be the protagonists of their learning process^(^ 43 ^)^. In addition, the curricular restructuring of the course is also important to provide the student with learning tools^(^ 44 ^-^ 45 ^)^ to complete the course on time^(^ 1 ^)^ and with appropriate educational support^(^ 13 ^)^, especially for students with learning difficulties.

In this sense, the opportunity to create integrated curricula for the education of nurses with clinical reasoning, critical thinking, and leadership skills with a focus on advanced nursing practice is seen in curriculum restructuring^(^ 46 ^)^, in view of its importance for individual and social transformation based on the active participation of the users of the Unified Health System (*Sistema Único de Saúde*, SUS)^(^ 47 ^)^ and possible future implementation in the national context^(^ 48 ^)^.

Given the undeniable need for nurses to be available in the labor market, especially associated with the excess and complexity of demands in the health services^(^ 49 ^)^, it is necessary to analyze disapproval as a structural phenomenon of retention and develop strategies for its mitigation in order to fully prepare more professionals in the time provided to fill up this gap, guaranteeing the high quality standards required for the profession.

The Brazilian scientific production on academic disapproval in nursing education is still deficient, highlighting the pioneering nature of this research. Thus, the present study can contribute to the improvement of strategies in the formulation of undergraduate curricula in nursing, aiming to avoid outcomes that negatively impact courses such as dropping out due to disapprovals, retention in the curriculum flow, cost and training time.

This study had as its main limitation the non-sampling stratification by period of the course: difficult to operate precisely because of the disapprovals generating non-uniform academic flows. Despite this, the study allowed for a wide analysis throughout the course of the occurrence of disapprovals with homogeneous distribution among the participants *per* year of entry into the course.

## Conclusion

In this study, a high frequency of disapprovals was observed among nursing students, with students aged 22 years old or over, family income below 2 minimum wages, and lag in the curriculum flow being associated with disapprovals in their academic path. The disciplines with the highest frequency of disapprovals are taught in the first two years of the course and are common to courses in the health area, namely: Pathology, Biochemistry, Physiology, Pharmacology and Histology, representing a dropout risk before the student gets to know the course better.

There was no difference in academic adaptation between students with or without failing; however, students without disapprovals had a better perception of physical and psychological well-being, good interpersonal relationships with peers, and assertive study behaviors. Keeping pace with classmates, good time management, keeping up with academic work, setting priorities, and having effective preparation for exams were protective behaviors against disapprovals.

As for the factors that motivate disapprovals, from the perspective of the participants, issues of a personal nature were pointed out: immaturity, the fact of reconciling study with work, and psychosomatic problems; issues related to study: learning difficulties, insufficient dedication, and difficulty in managing time; and institutional issues: professor-student relationship, methodology adopted by the professor in the discipline, and curricular structure.

The results suggest the need for investment in psycho-pedagogical support policies for students who enter nursing undergraduate courses, especially considering the change in sociodemographic profile and the difficulty arising from previous teaching. In addition, the need for constant review on the pedagogical projects of the course is reinforced, constantly improving the curricular structure with the possibility of articulating disciplines in order to avoid retentions due to prerequisite locks.

Future studies can be carried out in order to associate disapproval, retention and dropout with variables addressed in this study and others in a multi-factorial perspective, reinforcing the relevance of improving the education of nurses in Brazil.
